# Metabolic acidosis as Food Protein Induced Enterocolitis Syndrome (FPIES) onset in a newborn

**DOI:** 10.1186/s13052-018-0468-y

**Published:** 2018-05-10

**Authors:** Antonella Peduto, Mario Rocca, Cinzia De Maio, Federica Gallarotti, Giulia Pomero, Paolo Gancia

**Affiliations:** 1 Pediatric Unit, S. Croce Hospital, Cuneo, Italy; 2 Neonatal Intensive Care Unit, S. Croce Hospital, Cuneo, Italy

## Abstract

**Background:**

FPIES (Food Protein Induced Eneterolitis Syndrome) is a rare non IgE- mediated food allergy, usually affecting infants and children after first months of life. Clinical presentation is heterogeneous, usually characterised by repetitive vomiting and diarrhoea, lethargy, failure to thrive until to dehydration with hypotension and shock. The diagnosis is based on clinical criteria, after excludind other hypothetical conditions. Early recognition of FPIES is essential to set a correct dietatay management that is resolving for the patient.

**Case report:**

We present the case of a 12 days old child who was admitted to the hospital for poor feeding, failure to thrive and severe metabolic acidosis.

**Conclusions:**

The early onset of this case is peculiar and rember us to consider FPIES in differential diagnosis of newborn metabolica acidosis.

## Background

Food Protein Induced Enterocolitis Syndrome (FPIES) is an uncommon and potentially severe form of non IgE-mediated food allergy, that usually presents with profuse vomiting often associated to diarrhoea lethargy, pallor, dehydratation [1]. FPIES pathophysiology is not well known, it is hypothised an abnormal cell-mediated immunological disorder of gastrointestinal mucose after the ingestion of a trigger food, often represented by cow’s milk or soybean formula, potentially any food (fish, egg, wheat, rice, oat, meat, fruit and vegetables [2, 3]. In chronic cases, symptoms may include persistent diarrhoea, poor weight gain, failure to thrive, and improvement may be seen several days after the food elimination, sometimes requiring steroids treatment [4]. Age of onset of FPIES is variable, requiring a free-window time of allergen food exposure ranging from some weeks, usually after the first month of life or more, isolated cases of FPIES with adult onset have been reported [5]. Interestingly, a newborn with atypical presentation, ematochezia before the first feeding, was supposed to have acquired FPIES after sensibilization in the fetal period [6]. We describe a newborn who initially presented with misdiagnosed symptoms of dehydratation, lethargy, failure to thrive and severe metabolic acidosis.

## Case presentation

A 12 days old infant was admitted to Paediatric Emergency care after the evidence of weight lost since birth: –435 g corresponding to 12% from birth weight. He was born at tem by caesarean section, with birth weight of 3590 g, physiological perinatal course and normal clinical examination except for slight heart murmur corresponding to cardiac atrial-septal defect. He was discharged from hospital at 4 day of life with exclusive breastfeeding and weight 3296 g. At home the infant was fed with both breast milk and formula milk. During the last days before readmission the mother observed poor feeding with lethargy and frequently stool evacuations during the day. When the patient arrived in the hospital he was awake, fairly responsive with a preserved muscle tone, there were not evident signs of cardiovascular failure. Laboratory findings showed leukocytosis (WC 14190/ul, N 48%, L 41%, EOS 2,6%) with modest thrombocytosis (491.000/ul), important metabolic acidosis: pH 7,23, pCO2: 19 mmHg, HCO3: 8 mmol/l, base excess − 18.2 mmol/l. The infant was hospitalised in the Newborn Intensive care: intravenous glucose fluid and bicarbonate correction were started. Extensive investigations were done, considering lethargy and dehydratation in a critical newborn patient, sepsis was taken into account as one of the most frequent causes, but was ruled out by normal findings of infection markers (C-reactive protein level, bacterial cultures in biological samples). In the hypothesis of an inborn error of metabolism leading to organic acidemia, milk feeding was temporally suspended and an enteral nutrition with glicolipid formula was temporally started. At first laboratory detections, ketone bodies, ammonia, glycemia,, methemoglobinemia concentration and lactic acid were normal and an inborn error of metabolism was definitely excluded by the study of plasma amino acid profile and urine organic acids. In this case, metabolic acidosis was likely due to loss of bicarbonates from the gastrointestinal tract with the several diarrheal stools. The search of fecal blood, as other inflammatory index of gastrointestinal tract as calprotectin were negative, unfortunately we were not able to perform eosinophilic cells stool search. Even if the severity of clinical conditions could be suggestive, there were no elements for supposing necrotizing enterocolitis. On the basis of the clinical dates and in exclusion of other diagnosis, we assumed the hypothesis of milk protein induced non IgE-mediated allergy (specific milk protein IgE were negative). According to this diagnosis we changed patient’s diet with an amino acid formula milk, instead of extensive hydrolyzed formula, considering the clinical picture of malabsorption syndrome for prolonged diarrhoea. In few days we assisted to a considerable improvement of the infant’s clinical conditions with weight gain, normalisation of stools and consequently normalisation of acidosis.

After 2 months of selective diet the infant was readmitted to hospital to perform a diagnostic oral cow formula challenge, after parent’s consent was given. A peripheral intravenous line was placed and under medical supervision small cow-milk formula amounts were given to the infant by bottle. We started with 0.05 g of formula per kilogram of body weight and after 30 min we administered 0.2 g of milk per kilogram of body weight. After 3 h from milk ingestion the patient showed pallor and poor responsiveness, then he started vomiting (two episodes) and showed liquid stools within the next 10 h. Laboratory findings pointed out only moderate leukocytosis (WC: 12.100/ul), while cow milk specific IgE were still negative at the control. The observed reaction to the provocation test was diagnostic for FPIES-food protein induced enterocolitis syndrome.

## Discussion and conclusions

Since the first description of FPIES by Powell [7] several reports were made with an apparently increasing awareness regarding this rare condition. The incidence of FPIES is yet not well established, but it seems to be under diagnosed and often delayed from the beginning of symptoms. The difficulty in diagnosis mostly derives from the heterogeneity and not specificity of symptoms and from the lack of pathognomonic laboratory analysis [8]. Clinical criteria, suggested by Miceli Sopo et al. [9], for FPIES diagnosis in the acute form are the appearance of repetitive vomiting, pallor and lethargy within 2–4 h after the exposure to the trigger food; the resolution of illness and its recurrence within 2–4 h respectively with the avoidance and the reintroduction of the offending food. The oral food challenge (OFC) represents the only diagnostic test and it is recommended in all doubtful cases [10], above all when clinical picture is established “chronically” with intermittent vomiting, diarrhoea, failure to thrive and/or bloody stools while the infant is fed with milk or soy formulas in the first months of life. The OFC is usually done after a protein induced free time diet, under physician supervision because of the risk of severe allergic reaction.

The peculiarity of our case is the sneaky and very early presentation, nearly since birth and the absence of vomiting. Actually, although the mechanisms of occurrence have not been shown, FPIES is regarded as food protein stimulation of T-cells in the gastrointestinal mucosa, resulting in gastrointestinal inflammation mediated by various cytokines, such as TNF-α (tumor necrosis factor) and interleukin-6 [11]. For FPIES infants who present clinical symptoms at birth, the sensitization to milk protein could happen in the fetal period through the passage of allergenic proteins by umbilical cord blood [12].

In conclusion, early recognition of FPIES remains a critical issue to prevent misinterpretation and mistreatment of symptoms, especially when the clinical picture is severe like in neonates with metabolic acidosis as the main sign.
